# Non-merohedrally twinned hexa­methyl­enetetra­mine–4-nitro­phenol–water (1/2/1), triclinic modification

**DOI:** 10.1107/S1600536808034442

**Published:** 2008-10-25

**Authors:** Seik Weng Ng

**Affiliations:** a Department of Chemistry, University of Malaya, 50603 Kuala Lumpur, Malaysia

## Abstract

The asymmetric unit of the title cocrystal, C_6_H_12_N_4_·2C_6_H_5_NO_3_·H_2_O, contains four formula units, which are linked by O—H⋯O and O—H⋯N hydrogen bonds into chains in the crystal. The crystal studied was a non-merohedral twin, with a minor twin component of 19%.

## Related literature

For the background to this study and the *C*2 modification, see: Ng *et al.* (2001 ▶). The *P*1 modification is a non-merohedral twin; for the treatment of twinned diffraction data, see: Spek (2003 ▶).
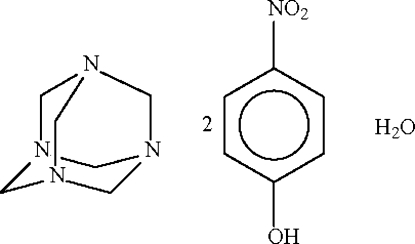


## Experimental

### 

#### Crystal data

C_6_H_12_N_4_·2C_6_H_5_NO_3_·H_2_O
*M* *_r_* = 436.43
Triclinic, 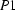

*a* = 6.9325 (1) Å
*b* = 11.6867 (2) Å
*c* = 25.0826 (5) Å
α = 96.728 (1)°
β = 92.449 (1)°
γ = 89.971 (1)°
*V* = 2016.29 (6) Å^3^
*Z* = 4
Mo *K*α radiation
μ = 0.11 mm^−1^
*T* = 100 (2) K
0.29 × 0.18 × 0.03 mm

#### Data collection

Bruker SMART APEX diffractometer
Absorption correction: none
32727 measured reflections
9162 independent reflections
7405 reflections with *I* > 2σ(*I*)
*R*_int_ = 0.045

#### Refinement

*R*[*F* ^2^ > 2σ(*F* ^2^)] = 0.079
*wR*(*F* ^2^) = 0.232
*S* = 1.06
9162 reflections
1118 parameters
27 restraints
H-atom parameters constrained
Δρ_max_ = 0.54 e Å^−3^
Δρ_min_ = −0.59 e Å^−3^

### 

Data collection: *APEX2* (Bruker, 2007 ▶); cell refinement: *SAINT* (Bruker, 2007 ▶); data reduction: *SAINT*; program(s) used to solve structure: *SHELXS97* (Sheldrick, 2008 ▶); program(s) used to refine structure: *SHELXL97* (Sheldrick, 2008 ▶); molecular graphics: *X-SEED* (Barbour, 2001 ▶); software used to prepare material for publication: *publCIF* (Westrip, 2008 ▶).

## Supplementary Material

Crystal structure: contains datablocks global, I. DOI: 10.1107/S1600536808034442/lh2700sup1.cif

Structure factors: contains datablocks I. DOI: 10.1107/S1600536808034442/lh2700Isup2.hkl

Additional supplementary materials:  crystallographic information; 3D view; checkCIF report

## Figures and Tables

**Table 1:** Hydrogen-bond geometry (Å, °)

*D*—H⋯*A*	*D*—H	H⋯*A*	*D*⋯*A*	*D*—H⋯*A*
O1—H1*o*⋯O1*w*	0.84	1.75	2.574 (7)	169
O4—H4*o*⋯O2*w*	0.84	1.76	2.585 (7)	168
O7—H7*o*⋯O3*w*	0.84	1.76	2.584 (7)	168
O10—H10*o*⋯O4*w*	0.84	1.76	2.584 (7)	168
O13—H13*o*⋯N2	0.84	1.81	2.649 (6)	173
O16—H16*o*⋯N6	0.84	1.81	2.641 (6)	170
O19—H19*o*⋯N10	0.84	1.82	2.648 (7)	170
O22—H22*o*⋯N14	0.84	1.81	2.645 (7)	171
O1*w*—H1*w*1⋯N1	0.84	2.01	2.814 (8)	161
O1*w*—H1*w*2⋯N4^i^	0.84	2.02	2.851 (7)	169
O2*w*—H2*w*1⋯N5	0.84	2.02	2.837 (7)	166
O2*w*—H2*w*2⋯N7^ii^	0.84	1.98	2.811 (7)	172
O3*w*—H3*w*1⋯N9	0.84	2.04	2.830 (8)	158
O3*w*—H3*w*2⋯N11^ii^	0.84	2.01	2.845 (7)	170
O4*w*—H4*w*1⋯N13	0.84	2.03	2.832 (7)	161
O4*w*—H4*w*2⋯N16^i^	0.84	1.99	2.819 (7)	171

Symmetry codes: (i) 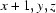; (ii) 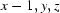.

**Fig. 1.**
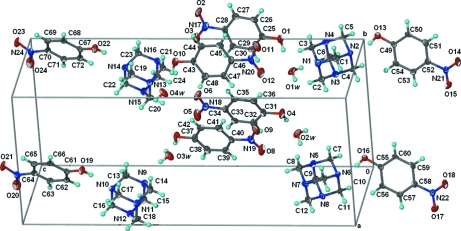
Thermal ellipsoid plot (Barbour, 2001) of the noncentrosymmetric, triclinic unit-cell contents of C6H12N4.2C6H5NO3.H2O. The probability level is 70%; H-atoms are shown as spheres of arbrary radius.

## supplementary crystallographic information

### Comment

The literature reports that the reaction of colorless hexamethylenetetramine and faint-yellow 4-nitrophenol in water/acetone yields deep yellow crystals. Two triclinic modifcations have been reported along with their unit cell cell constants [cell dimensions are 5.899 (1), 7.056 (1), 25.185 (11) Å, 87.53 (3), 96.71 (3), 90.0 (2)° for one polymorph and 11.781 (5), 7.046 (1), 25.174 (9) Å, 87.53 (2), 96.69 (3), 89.98 (2)° for the other polymorph]. In our studies, we found that the first polymorph is, in fact, a monoclinic *C*2 compound [49.989 (4), 5.9014 (5), 7.0556 (6) Å, β = 92.432 (2) °] (Ng *et al.*, 2001). However, although we were able to confirm the triclinic unit-cell dimensions of the second polymorph, we were not able to determine its crystal structure from the room-temperature measurements.

In the present study, we identified the title compound in space group *P*1 (Fig. 1), which also exists as plates, similar to the *C*2 compound (whose unit-cell volume at low temperature is not significantly different from that at room temperature). The triclinic polymorph has a significantly smaller volume [2016.29 (6) Å^3^] than the monoclinic polymorph [2079.6 (3) Å^3^], and is consequently denser (density 1.438 kg *M*^-3^) than the monoclinic polymorph (density 1.394 kg *M*^-3^). There does not appear to be a crystallographic relationship between the two modifications other than for one long axis. The triclinic polymorph is a nonmerohedral twin, the twin component being 18.9%.

### Experimental

The compound was obtained as deep yellow plates from the reaction of hexamethylenetetramine and 4-nitrophenol in acetone-water (Ng *et al.*, 2001).

### Refinement

The carbon- and oxygen-bound H-atoms were placed in calculated positions (C–H 0.95, O–H 0.84 Å) and were included in the refinement in the riding model approximation, with *U*(H) set to 1.2–1.5*U*(C,*O*). An *sp*^2^-type of hybridization was assumed for the hydroxy groups, *i.e.*, the phenolic H-atom is coplanar with the phenylene ring. Hydrogen atoms were placed on the phenolic oxygen atoms rather than on the ammonium nitrogen atoms as the compound displays hydroxyl absorption bands, as noted in the previous study (Ng *et al.*, 2001).

The water H-atoms were placed in chemically sensible positions on the basis of hydrogen bonding but were not refined; their temperature factors were similarly tied. The anisotropic temperature factors of the water O-atoms were restrained to be nearly isotropic. Of the eight H-atoms, four of them are about 2 Å from another H-atom; nevertheless, these four form unambigously short hydrogen bonds with acceptor atoms.

From the reflections used in the initial indexing of the unit cell, the *CELL_NOW* routine of the *APEX2* package (Bruker, 2007) had suggested the presence of a twin component, the routine giving the twin law as (-1 0 0, 0 - 1 0, -0.313 0 1). The routine also suggested that the minor twin component was rotated from the principal component by 179.8° about the reciprocal axis along (0 0 1). The structure initially refined to an *R* index of 9.1% when twinning was not considered.

The *TwinRotMat* routine in *PLATON* (Spek, 2003) gave the twin law as (1 0 0, 0 - 1 0, -1/3 0 - 1); 3001 reflections are overlapped. [The routine gave a second twin law (-1 0 0, 0 - 1 0, 0.309 0.503 1) with 1837 overlapping reflections.] The inclusion of the twin law in the refinement gave lowered the *R* index from 7.9%; the twin component refined to 18.9%. An examination of the standard deviations in bond distances showed that these were somewhat decreased. Both twin laws do not lead to a metrically larger monoclinic unit cell. For example, the first gave the cell as 6.933, 11.685, 25.086 Å, 96.73, 92.82, 90.04 °.

### Crystal data

**Table:**

C_6_H_12_N_4_·2C_6_H_5_NO_3_·H_2_O	*Z* = 4
*M**_r_* = 436.43	*F*(000) = 920
Triclinic, *P*1	*D*_x_ = 1.438 Mg m^−^^3^
Hall symbol: P 1	Mo *K*α radiation, λ = 0.71073 Å
*a* = 6.9325 (1) Å	Cell parameters from 8939 reflections
*b* = 11.6867 (2) Å	θ = 2.5–28.4°
*c* = 25.0826 (5) Å	µ = 0.11 mm^−^^1^
α = 96.728 (1)°	*T* = 100 K
β = 92.449 (1)°	Plate, yellow
γ = 89.971 (1)°	0.29 × 0.18 × 0.03 mm
*V* = 2016.29 (6) Å^3^	

### Data collection

**Table:**

Bruker SMART APEX diffractometer	7405 reflections with *I* > 2σ(*I*)
Radiation source: fine-focus sealed tube	*R*_int_ = 0.045
graphite	θ_max_ = 27.5°, θ_min_ = 0.8°
ω scans	*h* = −8→8
32727 measured reflections	*k* = −15→15
9162 independent reflections	*l* = −32→32

### Refinement

**Table:**

Refinement on *F*^2^	Primary atom site location: structure-invariant direct methods
Least-squares matrix: full	Secondary atom site location: difference Fourier map
*R*[*F*^2^ > 2σ(*F*^2^)] = 0.079	Hydrogen site location: inferred from neighbouring sites
*wR*(*F*^2^) = 0.232	H-atom parameters constrained
*S* = 1.06	*w* = 1/[σ^2^(*F*_o_^2^) + (0.1474*P*)^2^ + 2.0674*P*] where *P* = (*F*_o_^2^ + 2*F*_c_^2^)/3
9162 reflections	(Δ/σ)_max_ = 0.001
1118 parameters	Δρ_max_ = 0.54 e Å^−^^3^
27 restraints	Δρ_min_ = −0.59 e Å^−^^3^

### Fractional atomic coordinates and isotropic or equivalent isotropic displacement parameters (Å^2^)

**Table:**

	*x*	*y*	*z*	*U*_iso_*/*U*_eq_	
O1	0.0000 (8)	1.0000 (4)	0.25000 (18)	0.0288 (11)	
H1O	0.0238	0.9338	0.2349	0.043*	
O2	−0.0311 (8)	1.1938 (5)	0.4909 (2)	0.0323 (12)	
O3	0.0203 (9)	1.0152 (5)	0.4999 (2)	0.0368 (13)	
O4	0.1866 (8)	0.5010 (4)	0.24997 (18)	0.0284 (11)	
H4O	0.1581	0.4350	0.2347	0.043*	
O5	0.2458 (9)	0.5146 (5)	0.5001 (2)	0.0384 (13)	
O6	0.2985 (8)	0.6929 (5)	0.4906 (2)	0.0366 (13)	
O7	0.7337 (8)	0.5701 (4)	0.53257 (18)	0.0290 (11)	
H7O	0.7109	0.5113	0.5476	0.044*	
O8	0.7134 (9)	0.4609 (5)	0.2824 (2)	0.0362 (12)	
O9	0.7653 (8)	0.6442 (5)	0.2920 (2)	0.0334 (12)	
O10	0.5468 (8)	1.0711 (4)	0.53237 (18)	0.0276 (10)	
H10O	0.5746	1.0122	0.5474	0.041*	
O11	0.4365 (8)	1.1433 (5)	0.2920 (2)	0.0340 (12)	
O12	0.4867 (9)	0.9602 (5)	0.2824 (2)	0.0380 (13)	
O13	−0.4576 (7)	0.8156 (4)	−0.01168 (18)	0.0230 (10)	
H13O	−0.4352	0.7811	0.0155	0.034*	
O14	−0.3262 (8)	0.6738 (5)	−0.25474 (18)	0.0296 (11)	
O15	−0.2837 (8)	0.5090 (4)	−0.2236 (2)	0.0300 (11)	
O16	0.5311 (7)	0.3152 (4)	−0.01184 (17)	0.0203 (9)	
H16O	0.5524	0.2807	0.0154	0.031*	
O17	0.7228 (8)	0.0100 (4)	−0.2232 (2)	0.0300 (11)	
O18	0.6630 (8)	0.1727 (5)	−0.25473 (18)	0.0286 (11)	
O19	1.1932 (7)	0.5177 (4)	0.79411 (17)	0.0216 (9)	
H19O	1.1672	0.4706	0.7668	0.032*	
O20	1.0140 (8)	0.3173 (4)	1.0065 (2)	0.0314 (11)	
O21	1.0754 (7)	0.4958 (4)	1.03766 (18)	0.0275 (11)	
O22	0.2051 (7)	1.0164 (4)	0.79460 (17)	0.0211 (9)	
H22O	0.1847	0.9684	0.7673	0.032*	
O23	0.0583 (8)	0.9965 (4)	1.03681 (18)	0.0280 (11)	
O24	0.0167 (8)	0.8168 (5)	1.0063 (2)	0.0319 (11)	
O1W	0.0925 (7)	0.7915 (4)	0.2163 (2)	0.0270 (11)	
H1W1	−0.0025	0.7766	0.1949	0.040*	
H1W2	0.1951	0.7732	0.2009	0.040*	
O2W	0.0751 (7)	0.2933 (4)	0.21581 (19)	0.0262 (10)	
H2W1	0.1741	0.2618	0.2028	0.039*	
H2W2	−0.0222	0.2715	0.1960	0.039*	
O3W	0.6416 (7)	0.3774 (4)	0.5660 (2)	0.0265 (10)	
H3W1	0.7385	0.3778	0.5872	0.040*	
H3W2	0.5411	0.3681	0.5826	0.040*	
O4W	0.6586 (7)	0.8806 (4)	0.56655 (19)	0.0262 (10)	
H4W1	0.5582	0.8535	0.5776	0.039*	
H4W2	0.7536	0.8679	0.5869	0.039*	
N1	−0.2389 (8)	0.6989 (5)	0.1583 (2)	0.0203 (11)	
N2	−0.4161 (7)	0.6974 (4)	0.07192 (19)	0.0137 (9)	
N3	−0.4233 (7)	0.5292 (4)	0.1213 (2)	0.0174 (10)	
N4	−0.5907 (8)	0.7054 (4)	0.1549 (2)	0.0177 (10)	
N5	0.3858 (8)	0.2002 (4)	0.1548 (2)	0.0186 (10)	
N6	0.5578 (8)	0.1966 (4)	0.07150 (19)	0.0158 (10)	
N7	0.7398 (7)	0.2066 (4)	0.1580 (2)	0.0168 (10)	
N8	0.5663 (8)	0.0292 (4)	0.1221 (2)	0.0176 (10)	
N9	0.9764 (9)	0.3147 (5)	0.6242 (2)	0.0215 (11)	
N10	1.1517 (7)	0.3569 (4)	0.71079 (19)	0.0145 (9)	
N11	1.3260 (8)	0.3234 (4)	0.6276 (2)	0.0186 (11)	
N12	1.1612 (8)	0.1638 (4)	0.6617 (2)	0.0177 (10)	
N13	0.3488 (8)	0.8184 (4)	0.6275 (2)	0.0182 (10)	
N14	0.1774 (7)	0.8557 (4)	0.7111 (2)	0.0153 (10)	
N15	0.1693 (8)	0.6638 (4)	0.6604 (2)	0.0186 (10)	
N16	−0.0046 (7)	0.8238 (4)	0.6246 (2)	0.0161 (10)	
N17	−0.0069 (8)	1.0923 (5)	0.4722 (2)	0.0245 (12)	
N18	0.2681 (8)	0.5919 (5)	0.4719 (2)	0.0250 (12)	
N19	0.7411 (8)	0.5523 (6)	0.3108 (2)	0.0266 (12)	
N20	0.4658 (8)	1.0512 (5)	0.3104 (2)	0.0242 (12)	
N21	−0.3186 (7)	0.6122 (5)	−0.2173 (2)	0.0174 (10)	
N22	0.6797 (7)	0.1124 (5)	−0.2169 (2)	0.0175 (10)	
N23	1.0573 (7)	0.4161 (5)	0.9999 (2)	0.0183 (10)	
N24	0.0513 (7)	0.9164 (5)	0.9994 (2)	0.0172 (10)	
C1	−0.2423 (10)	0.7362 (5)	0.1042 (3)	0.0207 (12)	
H1A	−0.2354	0.8214	0.1076	0.031*	
H1B	−0.1271	0.7056	0.0855	0.031*	
C2	−0.2474 (9)	0.5718 (5)	0.1523 (2)	0.0174 (12)	
H2A	−0.1323	0.5403	0.1338	0.021*	
H2B	−0.2460	0.5448	0.1883	0.021*	
C3	−0.4100 (10)	0.7455 (5)	0.1854 (2)	0.0212 (13)	
H3A	−0.4046	0.8307	0.1892	0.025*	
H3B	−0.4105	0.7209	0.2219	0.025*	
C4	−0.4255 (10)	0.5694 (5)	0.0684 (2)	0.0182 (12)	
H4A	−0.5450	0.5418	0.0479	0.022*	
H4B	−0.3142	0.5361	0.0486	0.022*	
C5	−0.5887 (8)	0.7429 (5)	0.1016 (2)	0.0155 (11)	
H5A	−0.5872	0.8281	0.1050	0.019*	
H5B	−0.7079	0.7157	0.0808	0.019*	
C6	−0.5935 (11)	0.5792 (6)	0.1499 (3)	0.0252 (14)	
H6A	−0.7134	0.5505	0.1299	0.030*	
H6B	−0.5938	0.5536	0.1861	0.030*	
C7	0.3855 (9)	0.2376 (5)	0.1010 (2)	0.0178 (12)	
H7A	0.3818	0.3228	0.1043	0.021*	
H7B	0.2677	0.2079	0.0803	0.021*	
C8	0.5609 (9)	0.2481 (5)	0.1850 (2)	0.0188 (12)	
H8A	0.5635	0.2252	0.2218	0.023*	
H8B	0.5568	0.3333	0.1879	0.023*	
C9	0.3924 (10)	0.0751 (5)	0.1503 (3)	0.0204 (12)	
H9A	0.2747	0.0429	0.1303	0.024*	
H9B	0.3941	0.0500	0.1867	0.024*	
C10	0.7320 (8)	0.2417 (5)	0.1038 (2)	0.0165 (11)	
H10A	0.8488	0.2135	0.0851	0.020*	
H10B	0.7325	0.3269	0.1064	0.020*	
C11	0.5590 (9)	0.0687 (5)	0.0686 (2)	0.0165 (11)	
H11A	0.4414	0.0373	0.0483	0.020*	
H11B	0.6724	0.0384	0.0489	0.020*	
C12	0.7385 (9)	0.0797 (5)	0.1529 (3)	0.0197 (12)	
H12A	0.7425	0.0544	0.1893	0.024*	
H12B	0.8559	0.0505	0.1348	0.024*	
C13	0.9783 (9)	0.3790 (5)	0.6782 (2)	0.0201 (12)	
H13A	0.9705	0.4624	0.6749	0.024*	
H13B	0.8629	0.3573	0.6967	0.024*	
C14	1.1467 (10)	0.3480 (5)	0.5971 (2)	0.0207 (12)	
H14A	1.1409	0.4313	0.5933	0.025*	
H14B	1.1476	0.3052	0.5606	0.025*	
C15	0.9837 (9)	0.1904 (5)	0.6306 (2)	0.0195 (12)	
H15A	0.8690	0.1688	0.6494	0.023*	
H15B	0.9815	0.1446	0.5947	0.023*	
C16	1.1610 (9)	0.2318 (5)	0.7147 (2)	0.0167 (11)	
H16A	1.2797	0.2148	0.7357	0.020*	
H16B	1.0489	0.2087	0.7342	0.020*	
C17	1.3241 (9)	0.3878 (5)	0.6812 (2)	0.0160 (11)	
H17A	1.3223	0.4714	0.6779	0.019*	
H17B	1.4434	0.3711	0.7019	0.019*	
C18	1.3299 (11)	0.1996 (5)	0.6329 (3)	0.0256 (15)	
H18A	1.4504	0.1812	0.6527	0.031*	
H18B	1.3298	0.1556	0.5966	0.031*	
C19	0.3499 (10)	0.8824 (5)	0.6815 (2)	0.0205 (12)	
H19A	0.3535	0.9660	0.6784	0.025*	
H19B	0.4680	0.8628	0.7021	0.025*	
C20	0.3416 (10)	0.6948 (5)	0.6319 (3)	0.0221 (13)	
H20A	0.4597	0.6722	0.6516	0.026*	
H20B	0.3385	0.6516	0.5955	0.026*	
C21	0.1729 (9)	0.8512 (5)	0.5974 (2)	0.0177 (11)	
H21A	0.1696	0.8094	0.5606	0.021*	
H21B	0.1770	0.9348	0.5942	0.021*	
C22	0.1745 (9)	0.7298 (5)	0.7137 (2)	0.0178 (12)	
H22A	0.2909	0.7080	0.7345	0.021*	
H22B	0.0598	0.7097	0.7330	0.021*	
C23	0.0025 (8)	0.8851 (5)	0.6791 (2)	0.0161 (11)	
H23A	−0.1141	0.8656	0.6976	0.019*	
H23B	0.0014	0.9692	0.6768	0.019*	
C24	−0.0049 (10)	0.6986 (5)	0.6296 (3)	0.0213 (13)	
H24A	−0.0098	0.6549	0.5933	0.026*	
H24B	−0.1220	0.6789	0.6480	0.026*	
C25	−0.0037 (10)	1.0192 (6)	0.3040 (2)	0.0218 (12)	
C26	−0.0437 (8)	1.1300 (5)	0.3267 (3)	0.0179 (12)	
H26	−0.0698	1.1889	0.3043	0.022*	
C27	−0.0455 (9)	1.1545 (5)	0.3820 (3)	0.0192 (12)	
H27	−0.0693	1.2307	0.3979	0.023*	
C28	−0.0120 (9)	1.0666 (5)	0.4139 (2)	0.0182 (12)	
C29	0.0255 (9)	0.9538 (5)	0.3917 (2)	0.0181 (12)	
H29	0.0507	0.8948	0.4141	0.022*	
C30	0.0249 (9)	0.9302 (5)	0.3361 (3)	0.0206 (12)	
H30	0.0440	0.8536	0.3199	0.025*	
C31	0.2107 (10)	0.5189 (6)	0.3035 (2)	0.0211 (12)	
C32	0.1907 (9)	0.4298 (6)	0.3359 (3)	0.0217 (12)	
H32	0.1646	0.3533	0.3200	0.026*	
C33	0.2095 (9)	0.4548 (5)	0.3915 (2)	0.0179 (12)	
H33	0.1926	0.3959	0.4139	0.021*	
C34	0.2530 (8)	0.5658 (6)	0.4136 (2)	0.0180 (11)	
C35	0.2770 (8)	0.6543 (5)	0.3821 (2)	0.0161 (11)	
H35	0.3062	0.7303	0.3982	0.019*	
C36	0.2579 (8)	0.6301 (5)	0.3268 (2)	0.0167 (11)	
H36	0.2769	0.6894	0.3047	0.020*	
C37	0.7389 (9)	0.5622 (6)	0.4788 (2)	0.0193 (12)	
C38	0.7091 (9)	0.4570 (6)	0.4465 (2)	0.0212 (12)	
H38	0.6884	0.3884	0.4624	0.025*	
C39	0.7101 (8)	0.4539 (5)	0.3911 (2)	0.0164 (11)	
H39	0.6866	0.3836	0.3686	0.020*	
C40	0.7463 (9)	0.5554 (5)	0.3688 (2)	0.0173 (11)	
C41	0.7790 (9)	0.6587 (6)	0.4008 (3)	0.0203 (12)	
H41	0.8027	0.7270	0.3849	0.024*	
C42	0.7772 (8)	0.6619 (5)	0.4559 (3)	0.0180 (12)	
H42	0.8020	0.7322	0.4782	0.022*	
C43	0.5223 (10)	1.0629 (6)	0.4788 (2)	0.0209 (12)	
C44	0.4768 (8)	1.1615 (5)	0.4559 (2)	0.0171 (11)	
H44	0.4584	1.2318	0.4782	0.021*	
C45	0.4578 (8)	1.1589 (5)	0.4006 (2)	0.0168 (11)	
H45	0.4295	1.2271	0.3846	0.020*	
C46	0.4812 (9)	1.0541 (6)	0.3689 (2)	0.0201 (12)	
C47	0.5250 (8)	0.9543 (5)	0.3906 (2)	0.0179 (11)	
H47	0.5419	0.8843	0.3679	0.022*	
C48	0.5443 (9)	0.9572 (6)	0.4463 (2)	0.0214 (12)	
H48	0.5719	0.8887	0.4622	0.026*	
C49	−0.4198 (8)	0.7649 (5)	−0.0601 (2)	0.0140 (11)	
C50	−0.4591 (8)	0.8252 (5)	−0.1044 (2)	0.0165 (11)	
H50	−0.5127	0.9003	−0.0989	0.020*	
C51	−0.4212 (9)	0.7776 (5)	−0.1559 (2)	0.0173 (11)	
H51	−0.4445	0.8198	−0.1856	0.021*	
C52	−0.3481 (8)	0.6664 (5)	−0.1630 (2)	0.0155 (11)	
C53	−0.3059 (8)	0.6042 (5)	−0.1202 (2)	0.0149 (11)	
H53	−0.2558	0.5283	−0.1263	0.018*	
C54	−0.3381 (8)	0.6545 (5)	−0.0682 (2)	0.0155 (11)	
H54	−0.3049	0.6143	−0.0383	0.019*	
C55	0.5742 (8)	0.2650 (5)	−0.0603 (2)	0.0143 (11)	
C56	0.6575 (9)	0.1544 (5)	−0.0677 (2)	0.0161 (11)	
H56	0.6885	0.1141	−0.0377	0.019*	
C57	0.6938 (8)	0.1048 (5)	−0.1198 (2)	0.0157 (11)	
H57	0.7469	0.0296	−0.1257	0.019*	
C58	0.6512 (9)	0.1666 (5)	−0.1629 (2)	0.0170 (11)	
C59	0.5750 (8)	0.2779 (5)	−0.1556 (2)	0.0157 (11)	
H59	0.5504	0.3197	−0.1855	0.019*	
C60	0.5364 (8)	0.3255 (5)	−0.1046 (2)	0.0147 (11)	
H60	0.4832	0.4007	−0.0992	0.018*	
C61	1.1547 (8)	0.4908 (5)	0.8427 (2)	0.0136 (10)	
C62	1.0706 (8)	0.3851 (5)	0.8509 (2)	0.0152 (11)	
H62	1.0349	0.3304	0.8211	0.018*	
C63	1.0400 (9)	0.3610 (5)	0.9029 (2)	0.0167 (11)	
H63	0.9894	0.2883	0.9090	0.020*	
C64	1.0840 (9)	0.4442 (5)	0.9457 (2)	0.0172 (11)	
C65	1.1611 (9)	0.5519 (5)	0.9384 (2)	0.0177 (11)	
H65	1.1881	0.6084	0.9682	0.021*	
C66	1.1967 (8)	0.5737 (5)	0.8870 (2)	0.0149 (11)	
H66	1.2504	0.6459	0.8814	0.018*	
C67	0.1603 (8)	0.9904 (5)	0.8427 (2)	0.0152 (11)	
C68	0.1968 (8)	1.0732 (5)	0.8871 (2)	0.0143 (11)	
H68	0.2507	1.1455	0.8817	0.017*	
C69	0.1565 (8)	1.0522 (5)	0.9382 (2)	0.0165 (11)	
H69	0.1791	1.1094	0.9680	0.020*	
C70	0.0813 (9)	0.9442 (5)	0.9452 (2)	0.0169 (11)	
C71	0.0430 (8)	0.8595 (5)	0.9024 (2)	0.0144 (11)	
H71	−0.0067	0.7865	0.9083	0.017*	
C72	0.0790 (9)	0.8836 (5)	0.8507 (2)	0.0160 (11)	
H72	0.0486	0.8280	0.8208	0.019*	

### Atomic displacement parameters (Å^2^)

**Table:**

	*U*^11^	*U*^22^	*U*^33^	*U*^12^	*U*^13^	*U*^23^
O1	0.038 (3)	0.032 (3)	0.015 (2)	0.007 (2)	−0.0044 (19)	−0.0010 (18)
O2	0.034 (3)	0.038 (3)	0.021 (2)	0.000 (2)	−0.0021 (19)	−0.014 (2)
O3	0.052 (3)	0.044 (3)	0.016 (2)	0.005 (3)	0.002 (2)	0.009 (2)
O4	0.044 (3)	0.030 (3)	0.011 (2)	−0.005 (2)	0.0047 (19)	0.0012 (17)
O5	0.044 (3)	0.052 (4)	0.020 (2)	−0.008 (3)	0.000 (2)	0.012 (2)
O6	0.043 (3)	0.039 (3)	0.023 (3)	0.000 (2)	0.001 (2)	−0.015 (2)
O7	0.037 (3)	0.034 (3)	0.017 (2)	−0.008 (2)	−0.0012 (19)	0.0047 (19)
O8	0.043 (3)	0.047 (3)	0.018 (2)	−0.006 (3)	0.003 (2)	−0.001 (2)
O9	0.038 (3)	0.043 (3)	0.022 (2)	−0.001 (2)	0.001 (2)	0.020 (2)
O10	0.039 (3)	0.030 (2)	0.015 (2)	0.006 (2)	0.0028 (19)	0.0038 (18)
O11	0.035 (3)	0.046 (3)	0.026 (3)	−0.001 (2)	0.002 (2)	0.021 (2)
O12	0.042 (3)	0.049 (3)	0.020 (2)	0.006 (3)	0.000 (2)	−0.005 (2)
O13	0.035 (3)	0.018 (2)	0.015 (2)	0.0063 (18)	−0.0012 (18)	0.0024 (17)
O14	0.037 (3)	0.041 (3)	0.012 (2)	0.007 (2)	0.0015 (19)	0.0080 (19)
O15	0.038 (3)	0.028 (3)	0.021 (2)	0.011 (2)	0.000 (2)	−0.0076 (19)
O16	0.031 (3)	0.016 (2)	0.015 (2)	0.0058 (17)	−0.0016 (17)	0.0031 (16)
O17	0.039 (3)	0.027 (3)	0.022 (2)	0.010 (2)	−0.001 (2)	−0.0046 (19)
O18	0.034 (3)	0.040 (3)	0.013 (2)	0.006 (2)	0.0028 (18)	0.0068 (19)
O19	0.033 (3)	0.017 (2)	0.0142 (19)	−0.0049 (18)	0.0021 (18)	0.0011 (16)
O20	0.041 (3)	0.029 (3)	0.026 (2)	−0.010 (2)	0.005 (2)	0.012 (2)
O21	0.032 (3)	0.034 (3)	0.015 (2)	−0.007 (2)	0.0038 (19)	−0.0055 (19)
O22	0.029 (2)	0.019 (2)	0.015 (2)	−0.0065 (18)	0.0019 (17)	0.0026 (16)
O23	0.034 (3)	0.034 (3)	0.015 (2)	−0.005 (2)	0.0035 (19)	−0.0032 (19)
O24	0.041 (3)	0.031 (3)	0.025 (2)	−0.013 (2)	0.002 (2)	0.011 (2)
O1W	0.018 (2)	0.034 (3)	0.026 (2)	0.0048 (18)	0.0031 (18)	−0.0127 (19)
O2W	0.016 (2)	0.033 (3)	0.026 (2)	−0.0023 (18)	0.0024 (17)	−0.0124 (19)
O3W	0.018 (2)	0.039 (3)	0.027 (2)	−0.0014 (18)	0.0039 (18)	0.019 (2)
O4W	0.014 (2)	0.042 (3)	0.027 (2)	0.0012 (18)	0.0032 (17)	0.022 (2)
N1	0.026 (3)	0.017 (3)	0.017 (2)	0.000 (2)	−0.001 (2)	0.000 (2)
N2	0.019 (2)	0.010 (2)	0.012 (2)	0.0003 (17)	0.0041 (18)	0.0011 (17)
N3	0.021 (3)	0.013 (2)	0.019 (2)	−0.0008 (19)	0.0010 (19)	0.0029 (18)
N4	0.019 (3)	0.015 (2)	0.019 (2)	0.0030 (19)	0.0045 (19)	0.0017 (19)
N5	0.022 (3)	0.019 (3)	0.014 (2)	−0.002 (2)	0.0038 (19)	−0.0009 (19)
N6	0.023 (3)	0.012 (2)	0.013 (2)	0.0032 (18)	0.0036 (19)	0.0006 (17)
N7	0.013 (2)	0.017 (2)	0.020 (2)	−0.0024 (18)	−0.0020 (19)	0.0025 (19)
N8	0.022 (3)	0.014 (2)	0.016 (2)	0.0020 (19)	−0.0041 (19)	0.0047 (18)
N9	0.030 (3)	0.017 (3)	0.018 (2)	−0.002 (2)	−0.001 (2)	0.002 (2)
N10	0.016 (2)	0.013 (2)	0.015 (2)	−0.0003 (17)	0.0038 (18)	0.0019 (17)
N11	0.020 (3)	0.018 (3)	0.019 (2)	−0.0025 (19)	0.006 (2)	0.003 (2)
N12	0.023 (3)	0.011 (2)	0.020 (2)	−0.0057 (19)	0.003 (2)	0.0027 (18)
N13	0.020 (3)	0.018 (3)	0.017 (2)	0.001 (2)	0.004 (2)	0.0050 (19)
N14	0.019 (3)	0.012 (2)	0.015 (2)	−0.0016 (18)	0.0015 (18)	0.0027 (17)
N15	0.024 (3)	0.013 (2)	0.019 (2)	−0.0067 (19)	−0.001 (2)	0.0016 (19)
N16	0.011 (2)	0.019 (2)	0.017 (2)	−0.0007 (18)	−0.0027 (18)	0.0006 (19)
N17	0.019 (3)	0.034 (3)	0.018 (3)	−0.005 (2)	0.004 (2)	−0.005 (2)
N18	0.016 (3)	0.037 (3)	0.019 (3)	0.004 (2)	−0.002 (2)	−0.005 (2)
N19	0.022 (3)	0.042 (4)	0.018 (3)	0.007 (2)	0.002 (2)	0.009 (2)
N20	0.019 (3)	0.038 (3)	0.017 (3)	−0.003 (2)	−0.002 (2)	0.011 (2)
N21	0.014 (2)	0.026 (3)	0.011 (2)	0.0003 (19)	0.0010 (18)	0.0012 (19)
N22	0.014 (2)	0.026 (3)	0.013 (2)	0.0007 (19)	0.0017 (18)	0.0009 (19)
N23	0.014 (2)	0.024 (3)	0.018 (2)	−0.0007 (19)	0.0029 (19)	0.006 (2)
N24	0.014 (2)	0.023 (3)	0.015 (2)	−0.0040 (19)	−0.0002 (18)	0.0025 (19)
C1	0.024 (3)	0.017 (3)	0.022 (3)	−0.002 (2)	0.003 (2)	0.006 (2)
C2	0.021 (3)	0.014 (3)	0.017 (3)	0.002 (2)	0.003 (2)	0.003 (2)
C3	0.029 (3)	0.016 (3)	0.018 (3)	0.006 (2)	0.004 (2)	−0.001 (2)
C4	0.031 (3)	0.010 (3)	0.012 (3)	0.005 (2)	0.002 (2)	−0.002 (2)
C5	0.016 (3)	0.012 (3)	0.019 (3)	0.004 (2)	0.005 (2)	0.003 (2)
C6	0.031 (4)	0.019 (3)	0.028 (3)	−0.005 (3)	0.015 (3)	0.005 (2)
C7	0.023 (3)	0.016 (3)	0.015 (3)	0.006 (2)	0.000 (2)	0.003 (2)
C8	0.020 (3)	0.019 (3)	0.017 (3)	−0.005 (2)	0.001 (2)	0.000 (2)
C9	0.022 (3)	0.018 (3)	0.021 (3)	−0.010 (2)	0.003 (2)	0.002 (2)
C10	0.015 (3)	0.013 (3)	0.022 (3)	−0.006 (2)	0.005 (2)	0.001 (2)
C11	0.026 (3)	0.012 (3)	0.012 (2)	0.004 (2)	0.001 (2)	−0.001 (2)
C12	0.023 (3)	0.015 (3)	0.021 (3)	−0.003 (2)	−0.006 (2)	0.003 (2)
C13	0.023 (3)	0.018 (3)	0.019 (3)	0.002 (2)	0.002 (2)	0.000 (2)
C14	0.030 (3)	0.019 (3)	0.014 (3)	−0.005 (2)	0.003 (2)	0.007 (2)
C15	0.025 (3)	0.015 (3)	0.019 (3)	−0.004 (2)	0.004 (2)	0.002 (2)
C16	0.023 (3)	0.013 (3)	0.015 (3)	−0.002 (2)	0.000 (2)	0.006 (2)
C17	0.019 (3)	0.012 (3)	0.016 (3)	−0.003 (2)	0.001 (2)	0.000 (2)
C18	0.032 (4)	0.015 (3)	0.031 (3)	0.007 (2)	0.014 (3)	0.002 (3)
C19	0.026 (3)	0.016 (3)	0.020 (3)	−0.007 (2)	0.001 (2)	0.002 (2)
C20	0.027 (3)	0.017 (3)	0.022 (3)	0.007 (2)	0.006 (3)	0.003 (2)
C21	0.019 (3)	0.021 (3)	0.014 (3)	0.005 (2)	0.004 (2)	0.006 (2)
C22	0.026 (3)	0.012 (3)	0.016 (3)	−0.004 (2)	−0.002 (2)	0.005 (2)
C23	0.013 (3)	0.017 (3)	0.018 (3)	0.005 (2)	0.004 (2)	0.003 (2)
C24	0.024 (3)	0.015 (3)	0.024 (3)	−0.002 (2)	−0.005 (2)	−0.001 (2)
C25	0.023 (3)	0.027 (3)	0.016 (3)	0.007 (2)	0.000 (2)	0.006 (2)
C26	0.012 (3)	0.018 (3)	0.024 (3)	0.002 (2)	0.001 (2)	0.007 (2)
C27	0.017 (3)	0.016 (3)	0.023 (3)	−0.001 (2)	−0.002 (2)	0.000 (2)
C28	0.016 (3)	0.025 (3)	0.013 (3)	−0.002 (2)	0.001 (2)	0.001 (2)
C29	0.016 (3)	0.020 (3)	0.018 (3)	0.004 (2)	0.000 (2)	0.001 (2)
C30	0.019 (3)	0.018 (3)	0.024 (3)	0.006 (2)	0.000 (2)	0.000 (2)
C31	0.025 (3)	0.023 (3)	0.015 (3)	−0.003 (2)	−0.004 (2)	0.003 (2)
C32	0.021 (3)	0.024 (3)	0.021 (3)	−0.005 (2)	0.001 (2)	0.003 (2)
C33	0.015 (3)	0.017 (3)	0.022 (3)	0.003 (2)	0.002 (2)	0.006 (2)
C34	0.011 (3)	0.027 (3)	0.015 (3)	0.001 (2)	0.001 (2)	0.000 (2)
C35	0.010 (3)	0.013 (3)	0.025 (3)	−0.0014 (19)	0.000 (2)	−0.003 (2)
C36	0.011 (3)	0.020 (3)	0.020 (3)	0.003 (2)	0.003 (2)	0.005 (2)
C37	0.016 (3)	0.026 (3)	0.016 (3)	−0.008 (2)	−0.002 (2)	−0.001 (2)
C38	0.022 (3)	0.021 (3)	0.021 (3)	−0.007 (2)	−0.003 (2)	0.008 (2)
C39	0.009 (3)	0.019 (3)	0.020 (3)	−0.005 (2)	−0.001 (2)	0.001 (2)
C40	0.014 (3)	0.024 (3)	0.014 (3)	0.002 (2)	−0.002 (2)	0.005 (2)
C41	0.016 (3)	0.019 (3)	0.026 (3)	−0.001 (2)	−0.003 (2)	0.007 (2)
C42	0.012 (3)	0.016 (3)	0.026 (3)	−0.003 (2)	−0.001 (2)	0.000 (2)
C43	0.024 (3)	0.025 (3)	0.013 (3)	0.002 (2)	−0.003 (2)	0.002 (2)
C44	0.013 (3)	0.018 (3)	0.021 (3)	−0.003 (2)	0.003 (2)	0.003 (2)
C45	0.015 (3)	0.014 (3)	0.022 (3)	−0.002 (2)	0.003 (2)	0.008 (2)
C46	0.020 (3)	0.027 (3)	0.014 (3)	−0.001 (2)	0.001 (2)	0.004 (2)
C47	0.012 (3)	0.021 (3)	0.020 (3)	−0.003 (2)	−0.001 (2)	0.001 (2)
C48	0.024 (3)	0.021 (3)	0.019 (3)	0.001 (2)	0.003 (2)	0.002 (2)
C49	0.014 (3)	0.014 (3)	0.015 (2)	−0.003 (2)	−0.003 (2)	0.004 (2)
C50	0.014 (3)	0.013 (3)	0.024 (3)	0.002 (2)	−0.001 (2)	0.007 (2)
C51	0.015 (3)	0.021 (3)	0.016 (3)	−0.003 (2)	0.000 (2)	0.008 (2)
C52	0.013 (3)	0.018 (3)	0.016 (3)	−0.003 (2)	0.000 (2)	0.001 (2)
C53	0.016 (3)	0.012 (3)	0.016 (3)	−0.002 (2)	−0.001 (2)	0.000 (2)
C54	0.014 (3)	0.016 (3)	0.018 (3)	0.001 (2)	−0.001 (2)	0.006 (2)
C55	0.015 (3)	0.012 (3)	0.016 (3)	−0.001 (2)	−0.002 (2)	0.005 (2)
C56	0.015 (3)	0.013 (3)	0.021 (3)	0.002 (2)	−0.001 (2)	0.004 (2)
C57	0.017 (3)	0.012 (3)	0.018 (3)	0.001 (2)	0.001 (2)	0.000 (2)
C58	0.015 (3)	0.020 (3)	0.017 (3)	−0.001 (2)	0.001 (2)	0.004 (2)
C59	0.012 (3)	0.020 (3)	0.016 (3)	−0.002 (2)	−0.002 (2)	0.008 (2)
C60	0.010 (3)	0.013 (3)	0.022 (3)	0.0011 (19)	−0.003 (2)	0.006 (2)
C61	0.011 (2)	0.014 (3)	0.016 (3)	−0.001 (2)	−0.002 (2)	0.003 (2)
C62	0.017 (3)	0.011 (3)	0.017 (3)	0.000 (2)	−0.001 (2)	−0.001 (2)
C63	0.020 (3)	0.012 (3)	0.019 (3)	−0.004 (2)	0.002 (2)	0.003 (2)
C64	0.017 (3)	0.020 (3)	0.015 (3)	0.005 (2)	0.002 (2)	0.003 (2)
C65	0.015 (3)	0.015 (3)	0.022 (3)	0.002 (2)	−0.003 (2)	−0.002 (2)
C66	0.010 (3)	0.010 (2)	0.023 (3)	−0.0008 (19)	−0.003 (2)	−0.001 (2)
C67	0.013 (3)	0.014 (3)	0.017 (3)	−0.001 (2)	−0.006 (2)	0.002 (2)
C68	0.009 (3)	0.011 (2)	0.021 (3)	−0.0021 (19)	−0.004 (2)	−0.001 (2)
C69	0.012 (3)	0.015 (3)	0.021 (3)	0.002 (2)	−0.004 (2)	−0.003 (2)
C70	0.019 (3)	0.019 (3)	0.013 (3)	0.003 (2)	0.001 (2)	0.002 (2)
C71	0.016 (3)	0.009 (2)	0.018 (3)	−0.003 (2)	0.000 (2)	0.002 (2)
C72	0.019 (3)	0.013 (3)	0.016 (3)	−0.001 (2)	0.001 (2)	0.002 (2)

### Geometric parameters (Å, °)

**Table:**

O1—C25	1.347 (7)	C9—H9A	0.9900
O1—H1O	0.8400	C9—H9B	0.9900
O2—N17	1.238 (8)	C10—H10A	0.9900
O3—N17	1.212 (8)	C10—H10B	0.9900
O4—C31	1.338 (7)	C11—H11A	0.9900
O4—H4O	0.8400	C11—H11B	0.9900
O5—N18	1.225 (8)	C12—H12A	0.9900
O6—N18	1.233 (8)	C12—H12B	0.9900
O7—C37	1.343 (7)	C13—H13A	0.9900
O7—H7O	0.8400	C13—H13B	0.9900
O8—N19	1.223 (8)	C14—H14A	0.9900
O9—N19	1.236 (8)	C14—H14B	0.9900
O10—C43	1.340 (7)	C15—H15A	0.9900
O10—H10O	0.8400	C15—H15B	0.9900
O11—N20	1.233 (8)	C16—H16A	0.9900
O12—N20	1.216 (8)	C16—H16B	0.9900
O13—C49	1.325 (7)	C17—H17A	0.9900
O13—H13O	0.8400	C17—H17B	0.9900
O14—N21	1.248 (7)	C18—H18A	0.9900
O15—N21	1.223 (7)	C18—H18B	0.9900
O16—C55	1.332 (7)	C19—H19A	0.9900
O16—H16O	0.8400	C19—H19B	0.9900
O17—N22	1.227 (7)	C20—H20A	0.9900
O18—N22	1.247 (7)	C20—H20B	0.9900
O19—C61	1.332 (7)	C21—H21A	0.9900
O19—H19O	0.8400	C21—H21B	0.9900
O20—N23	1.226 (7)	C22—H22A	0.9900
O21—N23	1.251 (7)	C22—H22B	0.9900
O22—C67	1.327 (7)	C23—H23A	0.9900
O22—H22O	0.8400	C23—H23B	0.9900
O23—N24	1.244 (7)	C24—H24A	0.9900
O24—N24	1.222 (7)	C24—H24B	0.9900
O1W—H1W1	0.8369	C25—C26	1.385 (9)
O1W—H1W2	0.8393	C25—C30	1.397 (9)
O2W—H2W1	0.8385	C26—C27	1.384 (9)
O2W—H2W2	0.8420	C26—H26	0.9500
O3W—H3W1	0.8380	C27—C28	1.388 (9)
O3W—H3W2	0.8396	C27—H27	0.9500
O4W—H4W1	0.8366	C28—C29	1.398 (8)
O4W—H4W2	0.8399	C29—C30	1.390 (8)
N1—C3	1.465 (8)	C29—H29	0.9500
N1—C1	1.472 (8)	C30—H30	0.9500
N1—C2	1.475 (7)	C31—C36	1.394 (9)
N2—C1	1.465 (8)	C31—C32	1.404 (8)
N2—C4	1.489 (7)	C32—C33	1.390 (8)
N2—C5	1.500 (7)	C32—H32	0.9500
N3—C4	1.457 (7)	C33—C34	1.379 (9)
N3—C2	1.473 (8)	C33—H33	0.9500
N3—C6	1.490 (8)	C34—C35	1.388 (8)
N4—C5	1.456 (7)	C35—C36	1.382 (8)
N4—C6	1.465 (8)	C35—H35	0.9500
N4—C3	1.486 (9)	C36—H36	0.9500
N5—C9	1.455 (8)	C37—C42	1.388 (8)
N5—C7	1.466 (7)	C37—C38	1.401 (9)
N5—C8	1.477 (8)	C38—C39	1.385 (8)
N6—C7	1.482 (7)	C38—H38	0.9500
N6—C10	1.486 (8)	C39—C40	1.396 (8)
N6—C11	1.488 (7)	C39—H39	0.9500
N7—C10	1.465 (8)	C40—C41	1.382 (9)
N7—C12	1.474 (8)	C41—C42	1.380 (9)
N7—C8	1.491 (8)	C41—H41	0.9500
N8—C11	1.468 (7)	C42—H42	0.9500
N8—C12	1.478 (8)	C43—C44	1.377 (8)
N8—C9	1.492 (8)	C43—C48	1.410 (9)
N9—C14	1.463 (8)	C44—C45	1.384 (8)
N9—C13	1.469 (8)	C44—H44	0.9500
N9—C15	1.481 (8)	C45—C46	1.393 (9)
N10—C13	1.465 (8)	C45—H45	0.9500
N10—C16	1.479 (7)	C46—C47	1.371 (9)
N10—C17	1.500 (7)	C47—C48	1.396 (8)
N11—C17	1.461 (7)	C47—H47	0.9500
N11—C18	1.468 (8)	C48—H48	0.9500
N11—C14	1.477 (9)	C49—C50	1.401 (8)
N12—C16	1.467 (8)	C49—C54	1.405 (8)
N12—C15	1.480 (8)	C50—C51	1.380 (8)
N12—C18	1.486 (8)	C50—H50	0.9500
N13—C20	1.463 (8)	C51—C52	1.390 (8)
N13—C19	1.468 (8)	C51—H51	0.9500
N13—C21	1.481 (8)	C52—C53	1.386 (8)
N14—C22	1.481 (7)	C53—C54	1.395 (8)
N14—C19	1.487 (8)	C53—H53	0.9500
N14—C23	1.489 (8)	C54—H54	0.9500
N15—C22	1.464 (8)	C55—C60	1.401 (7)
N15—C20	1.483 (8)	C55—C56	1.411 (8)
N15—C24	1.487 (8)	C56—C57	1.400 (8)
N16—C23	1.465 (8)	C56—H56	0.9500
N16—C24	1.483 (8)	C57—C58	1.389 (8)
N16—C21	1.486 (7)	C57—H57	0.9500
N17—C28	1.455 (7)	C58—C59	1.398 (8)
N18—C34	1.457 (7)	C59—C60	1.372 (8)
N19—C40	1.451 (7)	C59—H59	0.9500
N20—C46	1.463 (7)	C60—H60	0.9500
N21—C52	1.455 (7)	C61—C62	1.407 (8)
N22—C58	1.449 (8)	C61—C66	1.407 (8)
N23—C64	1.454 (7)	C62—C63	1.391 (8)
N24—C70	1.458 (7)	C62—H62	0.9500
C1—H1A	0.9900	C63—C64	1.386 (8)
C1—H1B	0.9900	C63—H63	0.9500
C2—H2A	0.9900	C64—C65	1.403 (9)
C2—H2B	0.9900	C65—C66	1.375 (9)
C3—H3A	0.9900	C65—H65	0.9500
C3—H3B	0.9900	C66—H66	0.9500
C4—H4A	0.9900	C67—C68	1.402 (8)
C4—H4B	0.9900	C67—C72	1.409 (8)
C5—H5A	0.9900	C68—C69	1.374 (8)
C5—H5B	0.9900	C68—H68	0.9500
C6—H6A	0.9900	C69—C70	1.399 (8)
C6—H6B	0.9900	C69—H69	0.9500
C7—H7A	0.9900	C70—C71	1.390 (8)
C7—H7B	0.9900	C71—C72	1.390 (8)
C8—H8A	0.9900	C71—H71	0.9500
C8—H8B	0.9900	C72—H72	0.9500
			
C25—O1—H1O	120.0	N11—C18—N12	111.6 (5)
C31—O4—H4O	120.0	N11—C18—H18A	109.3
C37—O7—H7O	120.0	N12—C18—H18A	109.3
C43—O10—H10O	120.0	N11—C18—H18B	109.3
C49—O13—H13O	120.0	N12—C18—H18B	109.3
C55—O16—H16O	120.0	H18A—C18—H18B	108.0
C61—O19—H19O	120.0	N13—C19—N14	112.0 (5)
C67—O22—H22O	120.0	N13—C19—H19A	109.2
H1W1—O1W—H1W2	110.0	N14—C19—H19A	109.2
H2W1—O2W—H2W2	109.3	N13—C19—H19B	109.2
H3W1—O3W—H3W2	109.7	N14—C19—H19B	109.2
H4W1—O4W—H4W2	109.8	H19A—C19—H19B	107.9
C3—N1—C1	108.2 (5)	N13—C20—N15	111.5 (5)
C3—N1—C2	109.4 (5)	N13—C20—H20A	109.3
C1—N1—C2	108.1 (5)	N15—C20—H20A	109.3
C1—N2—C4	108.1 (5)	N13—C20—H20B	109.3
C1—N2—C5	108.1 (5)	N15—C20—H20B	109.3
C4—N2—C5	106.9 (4)	H20A—C20—H20B	108.0
C4—N3—C2	109.4 (5)	N13—C21—N16	111.3 (5)
C4—N3—C6	108.1 (5)	N13—C21—H21A	109.4
C2—N3—C6	108.1 (5)	N16—C21—H21A	109.4
C5—N4—C6	109.2 (5)	N13—C21—H21B	109.4
C5—N4—C3	108.7 (5)	N16—C21—H21B	109.4
C6—N4—C3	107.8 (5)	H21A—C21—H21B	108.0
C9—N5—C7	109.4 (5)	N15—C22—N14	112.2 (4)
C9—N5—C8	109.2 (5)	N15—C22—H22A	109.2
C7—N5—C8	107.7 (5)	N14—C22—H22A	109.2
C7—N6—C10	107.9 (4)	N15—C22—H22B	109.2
C7—N6—C11	107.1 (5)	N14—C22—H22B	109.2
C10—N6—C11	108.1 (5)	H22A—C22—H22B	107.9
C10—N7—C12	107.9 (5)	N16—C23—N14	112.2 (4)
C10—N7—C8	108.1 (5)	N16—C23—H23A	109.2
C12—N7—C8	107.9 (5)	N14—C23—H23A	109.2
C11—N8—C12	108.9 (5)	N16—C23—H23B	109.2
C11—N8—C9	107.6 (5)	N14—C23—H23B	109.2
C12—N8—C9	107.7 (5)	H23A—C23—H23B	107.9
C14—N9—C13	108.5 (5)	N16—C24—N15	111.8 (5)
C14—N9—C15	110.1 (5)	N16—C24—H24A	109.3
C13—N9—C15	107.7 (5)	N15—C24—H24A	109.3
C13—N10—C16	108.0 (5)	N16—C24—H24B	109.3
C13—N10—C17	107.9 (5)	N15—C24—H24B	109.3
C16—N10—C17	107.3 (4)	H24A—C24—H24B	107.9
C17—N11—C18	109.0 (5)	O1—C25—C26	117.6 (6)
C17—N11—C14	109.0 (5)	O1—C25—C30	121.6 (6)
C18—N11—C14	108.2 (5)	C26—C25—C30	120.8 (6)
C16—N12—C15	108.7 (5)	C27—C26—C25	119.7 (6)
C16—N12—C18	108.6 (5)	C27—C26—H26	120.1
C15—N12—C18	108.0 (5)	C25—C26—H26	120.1
C20—N13—C19	109.3 (5)	C26—C27—C28	119.3 (6)
C20—N13—C21	109.0 (5)	C26—C27—H27	120.3
C19—N13—C21	108.0 (5)	C28—C27—H27	120.3
C22—N14—C19	107.7 (5)	C27—C28—C29	121.8 (5)
C22—N14—C23	107.8 (5)	C27—C28—N17	119.7 (6)
C19—N14—C23	108.0 (5)	C29—C28—N17	118.5 (5)
C22—N15—C20	108.6 (5)	C30—C29—C28	118.3 (5)
C22—N15—C24	108.4 (5)	C30—C29—H29	120.9
C20—N15—C24	107.8 (5)	C28—C29—H29	120.9
C23—N16—C24	107.6 (5)	C29—C30—C25	120.0 (6)
C23—N16—C21	109.0 (5)	C29—C30—H30	120.0
C24—N16—C21	108.1 (5)	C25—C30—H30	120.0
O3—N17—O2	123.1 (6)	O4—C31—C36	117.8 (5)
O3—N17—C28	119.5 (6)	O4—C31—C32	122.1 (6)
O2—N17—C28	117.3 (6)	C36—C31—C32	120.2 (6)
O5—N18—O6	122.8 (6)	C33—C32—C31	119.3 (6)
O5—N18—C34	119.5 (6)	C33—C32—H32	120.3
O6—N18—C34	117.7 (6)	C31—C32—H32	120.3
O8—N19—O9	122.4 (6)	C34—C33—C32	119.4 (5)
O8—N19—C40	119.9 (6)	C34—C33—H33	120.3
O9—N19—C40	117.7 (6)	C32—C33—H33	120.3
O12—N20—O11	123.3 (6)	C33—C34—C35	121.9 (5)
O12—N20—C46	119.3 (6)	C33—C34—N18	119.1 (5)
O11—N20—C46	117.4 (6)	C35—C34—N18	119.0 (6)
O15—N21—O14	123.4 (5)	C36—C35—C34	119.0 (5)
O15—N21—C52	118.2 (5)	C36—C35—H35	120.5
O14—N21—C52	118.3 (5)	C34—C35—H35	120.5
O17—N22—O18	123.1 (5)	C35—C36—C31	120.2 (5)
O17—N22—C58	118.4 (5)	C35—C36—H36	119.9
O18—N22—C58	118.4 (5)	C31—C36—H36	119.9
O20—N23—O21	123.0 (5)	O7—C37—C42	117.8 (6)
O20—N23—C64	119.3 (5)	O7—C37—C38	121.6 (6)
O21—N23—C64	117.7 (5)	C42—C37—C38	120.5 (6)
O24—N24—O23	122.8 (5)	C39—C38—C37	119.5 (6)
O24—N24—C70	119.2 (5)	C39—C38—H38	120.3
O23—N24—C70	118.0 (5)	C37—C38—H38	120.3
N2—C1—N1	112.6 (5)	C38—C39—C40	119.0 (5)
N2—C1—H1A	109.1	C38—C39—H39	120.5
N1—C1—H1A	109.1	C40—C39—H39	120.5
N2—C1—H1B	109.1	C41—C40—C39	121.5 (5)
N1—C1—H1B	109.1	C41—C40—N19	119.7 (6)
H1A—C1—H1B	107.8	C39—C40—N19	118.8 (5)
N3—C2—N1	110.9 (5)	C42—C41—C40	119.5 (6)
N3—C2—H2A	109.5	C42—C41—H41	120.3
N1—C2—H2A	109.5	C40—C41—H41	120.3
N3—C2—H2B	109.5	C41—C42—C37	120.0 (6)
N1—C2—H2B	109.5	C41—C42—H42	120.0
H2A—C2—H2B	108.0	C37—C42—H42	120.0
N1—C3—N4	111.4 (5)	O10—C43—C44	118.1 (6)
N1—C3—H3A	109.3	O10—C43—C48	121.5 (6)
N4—C3—H3A	109.3	C44—C43—C48	120.4 (6)
N1—C3—H3B	109.3	C43—C44—C45	120.5 (6)
N4—C3—H3B	109.3	C43—C44—H44	119.8
H3A—C3—H3B	108.0	C45—C44—H44	119.8
N3—C4—N2	112.1 (4)	C44—C45—C46	118.5 (5)
N3—C4—H4A	109.2	C44—C45—H45	120.7
N2—C4—H4A	109.2	C46—C45—H45	120.7
N3—C4—H4B	109.2	C47—C46—C45	122.4 (5)
N2—C4—H4B	109.2	C47—C46—N20	118.7 (6)
H4A—C4—H4B	107.9	C45—C46—N20	118.8 (6)
N4—C5—N2	111.6 (5)	C46—C47—C48	119.0 (6)
N4—C5—H5A	109.3	C46—C47—H47	120.5
N2—C5—H5A	109.3	C48—C47—H47	120.5
N4—C5—H5B	109.3	C47—C48—C43	119.2 (6)
N2—C5—H5B	109.3	C47—C48—H48	120.4
H5A—C5—H5B	108.0	C43—C48—H48	120.4
N4—C6—N3	111.6 (5)	O13—C49—C50	118.3 (5)
N4—C6—H6A	109.3	O13—C49—C54	122.3 (5)
N3—C6—H6A	109.3	C50—C49—C54	119.4 (5)
N4—C6—H6B	109.3	C51—C50—C49	121.2 (5)
N3—C6—H6B	109.3	C51—C50—H50	119.4
H6A—C6—H6B	108.0	C49—C50—H50	119.4
N5—C7—N6	112.1 (5)	C50—C51—C52	118.3 (5)
N5—C7—H7A	109.2	C50—C51—H51	120.8
N6—C7—H7A	109.2	C52—C51—H51	120.8
N5—C7—H7B	109.2	C53—C52—C51	122.2 (5)
N6—C7—H7B	109.2	C53—C52—N21	118.7 (5)
H7A—C7—H7B	107.9	C51—C52—N21	119.1 (5)
N5—C8—N7	111.4 (5)	C52—C53—C54	119.1 (5)
N5—C8—H8A	109.3	C52—C53—H53	120.4
N7—C8—H8A	109.3	C54—C53—H53	120.4
N5—C8—H8B	109.3	C53—C54—C49	119.7 (5)
N7—C8—H8B	109.3	C53—C54—H54	120.1
H8A—C8—H8B	108.0	C49—C54—H54	120.1
N5—C9—N8	111.5 (5)	O16—C55—C60	118.2 (5)
N5—C9—H9A	109.3	O16—C55—C56	121.9 (5)
N8—C9—H9A	109.3	C60—C55—C56	119.9 (5)
N5—C9—H9B	109.3	C57—C56—C55	119.2 (5)
N8—C9—H9B	109.3	C57—C56—H56	120.4
H9A—C9—H9B	108.0	C55—C56—H56	120.4
N7—C10—N6	112.5 (4)	C58—C57—C56	119.2 (5)
N7—C10—H10A	109.1	C58—C57—H57	120.4
N6—C10—H10A	109.1	C56—C57—H57	120.4
N7—C10—H10B	109.1	C57—C58—C59	121.9 (5)
N6—C10—H10B	109.1	C57—C58—N22	118.9 (5)
H10A—C10—H10B	107.8	C59—C58—N22	119.1 (5)
N8—C11—N6	112.1 (4)	C60—C59—C58	118.7 (5)
N8—C11—H11A	109.2	C60—C59—H59	120.7
N6—C11—H11A	109.2	C58—C59—H59	120.7
N8—C11—H11B	109.2	C59—C60—C55	121.1 (5)
N6—C11—H11B	109.2	C59—C60—H60	119.5
H11A—C11—H11B	107.9	C55—C60—H60	119.5
N7—C12—N8	112.6 (5)	O19—C61—C62	122.7 (5)
N7—C12—H12A	109.1	O19—C61—C66	117.8 (5)
N8—C12—H12A	109.1	C62—C61—C66	119.6 (5)
N7—C12—H12B	109.1	C63—C62—C61	119.7 (5)
N8—C12—H12B	109.1	C63—C62—H62	120.1
H12A—C12—H12B	107.8	C61—C62—H62	120.1
N10—C13—N9	112.7 (5)	C64—C63—C62	119.3 (5)
N10—C13—H13A	109.1	C64—C63—H63	120.4
N9—C13—H13A	109.1	C62—C63—H63	120.4
N10—C13—H13B	109.1	C63—C64—C65	122.0 (5)
N9—C13—H13B	109.1	C63—C64—N23	118.5 (5)
H13A—C13—H13B	107.8	C65—C64—N23	119.4 (5)
N9—C14—N11	111.0 (5)	C66—C65—C64	118.4 (5)
N9—C14—H14A	109.4	C66—C65—H65	120.8
N11—C14—H14A	109.4	C64—C65—H65	120.8
N9—C14—H14B	109.4	C65—C66—C61	121.0 (5)
N11—C14—H14B	109.4	C65—C66—H66	119.5
H14A—C14—H14B	108.0	C61—C66—H66	119.5
N12—C15—N9	110.4 (5)	O22—C67—C68	118.0 (5)
N12—C15—H15A	109.6	O22—C67—C72	122.9 (5)
N9—C15—H15A	109.6	C68—C67—C72	119.1 (5)
N12—C15—H15B	109.6	C69—C68—C67	121.5 (5)
N9—C15—H15B	109.6	C69—C68—H68	119.3
H15A—C15—H15B	108.1	C67—C68—H68	119.3
N12—C16—N10	112.1 (4)	C68—C69—C70	118.2 (5)
N12—C16—H16A	109.2	C68—C69—H69	120.9
N10—C16—H16A	109.2	C70—C69—H69	120.9
N12—C16—H16B	109.2	C71—C70—C69	122.3 (5)
N10—C16—H16B	109.2	C71—C70—N24	118.3 (5)
H16A—C16—H16B	107.9	C69—C70—N24	119.3 (5)
N11—C17—N10	111.4 (5)	C70—C71—C72	118.7 (5)
N11—C17—H17A	109.3	C70—C71—H71	120.6
N10—C17—H17A	109.3	C72—C71—H71	120.6
N11—C17—H17B	109.3	C71—C72—C67	120.2 (5)
N10—C17—H17B	109.3	C71—C72—H72	119.9
H17A—C17—H17B	108.0	C67—C72—H72	119.9
			
C4—N2—C1—N1	−57.6 (6)	O2—N17—C28—C29	176.5 (6)
C5—N2—C1—N1	57.7 (6)	C27—C28—C29—C30	−1.3 (9)
C3—N1—C1—N2	−59.0 (6)	N17—C28—C29—C30	−178.5 (5)
C2—N1—C1—N2	59.4 (7)	C28—C29—C30—C25	2.9 (9)
C4—N3—C2—N1	58.9 (6)	O1—C25—C30—C29	178.2 (6)
C6—N3—C2—N1	−58.5 (6)	C26—C25—C30—C29	−4.1 (10)
C3—N1—C2—N3	58.8 (6)	O4—C31—C32—C33	−177.3 (6)
C1—N1—C2—N3	−58.8 (6)	C36—C31—C32—C33	3.0 (10)
C1—N1—C3—N4	58.8 (6)	C31—C32—C33—C34	−1.9 (9)
C2—N1—C3—N4	−58.7 (6)	C32—C33—C34—C35	0.6 (9)
C5—N4—C3—N1	−59.5 (6)	C32—C33—C34—N18	178.6 (5)
C6—N4—C3—N1	58.8 (6)	O5—N18—C34—C33	1.4 (8)
C2—N3—C4—N2	−57.9 (6)	O6—N18—C34—C33	−176.8 (6)
C6—N3—C4—N2	59.6 (6)	O5—N18—C34—C35	179.5 (6)
C1—N2—C4—N3	56.6 (6)	O6—N18—C34—C35	1.2 (8)
C5—N2—C4—N3	−59.5 (6)	C33—C34—C35—C36	−0.4 (9)
C6—N4—C5—N2	−59.1 (6)	N18—C34—C35—C36	−178.4 (5)
C3—N4—C5—N2	58.3 (6)	C34—C35—C36—C31	1.5 (8)
C1—N2—C5—N4	−57.4 (6)	O4—C31—C36—C35	177.5 (5)
C4—N2—C5—N4	58.7 (6)	C32—C31—C36—C35	−2.8 (9)
C5—N4—C6—N3	58.5 (7)	O7—C37—C38—C39	178.1 (6)
C3—N4—C6—N3	−59.5 (7)	C42—C37—C38—C39	−2.6 (9)
C4—N3—C6—N4	−58.4 (7)	C37—C38—C39—C40	1.8 (9)
C2—N3—C6—N4	59.9 (7)	C38—C39—C40—C41	−0.6 (9)
C9—N5—C7—N6	−58.5 (6)	C38—C39—C40—N19	−178.1 (5)
C8—N5—C7—N6	60.1 (6)	O8—N19—C40—C41	179.5 (6)
C10—N6—C7—N5	−58.5 (6)	O9—N19—C40—C41	−0.6 (8)
C11—N6—C7—N5	57.8 (6)	O8—N19—C40—C39	−3.0 (9)
C9—N5—C8—N7	58.8 (6)	O9—N19—C40—C39	176.9 (6)
C7—N5—C8—N7	−60.0 (6)	C39—C40—C41—C42	0.3 (9)
C10—N7—C8—N5	59.0 (6)	N19—C40—C41—C42	177.8 (5)
C12—N7—C8—N5	−57.4 (6)	C40—C41—C42—C37	−1.2 (9)
C7—N5—C9—N8	58.3 (6)	O7—C37—C42—C41	−178.3 (6)
C8—N5—C9—N8	−59.3 (6)	C38—C37—C42—C41	2.4 (9)
C11—N8—C9—N5	−58.7 (6)	O10—C43—C44—C45	177.3 (6)
C12—N8—C9—N5	58.6 (6)	C48—C43—C44—C45	−1.9 (9)
C12—N7—C10—N6	58.7 (6)	C43—C44—C45—C46	1.5 (9)
C8—N7—C10—N6	−57.8 (6)	C44—C45—C46—C47	−1.0 (9)
C7—N6—C10—N7	57.4 (6)	C44—C45—C46—N20	−178.6 (5)
C11—N6—C10—N7	−58.2 (6)	O12—N20—C46—C47	2.1 (9)
C12—N8—C11—N6	−56.9 (6)	O11—N20—C46—C47	−176.2 (6)
C9—N8—C11—N6	59.6 (6)	O12—N20—C46—C45	179.8 (6)
C7—N6—C11—N8	−59.2 (6)	O11—N20—C46—C45	1.4 (8)
C10—N6—C11—N8	56.9 (6)	C45—C46—C47—C48	0.9 (9)
C10—N7—C12—N8	−58.3 (6)	N20—C46—C47—C48	178.5 (5)
C8—N7—C12—N8	58.3 (6)	C46—C47—C48—C43	−1.3 (9)
C11—N8—C12—N7	57.8 (6)	O10—C43—C48—C47	−177.4 (6)
C9—N8—C12—N7	−58.7 (6)	C44—C43—C48—C47	1.8 (10)
C16—N10—C13—N9	−58.2 (6)	O13—C49—C50—C51	179.3 (5)
C17—N10—C13—N9	57.5 (6)	C54—C49—C50—C51	0.8 (9)
C14—N9—C13—N10	−59.1 (6)	C49—C50—C51—C52	1.9 (9)
C15—N9—C13—N10	60.0 (7)	C50—C51—C52—C53	−2.4 (9)
C13—N9—C14—N11	59.0 (6)	C50—C51—C52—N21	176.2 (5)
C15—N9—C14—N11	−58.6 (6)	O15—N21—C52—C53	10.0 (8)
C17—N11—C14—N9	−59.7 (6)	O14—N21—C52—C53	−168.5 (5)
C18—N11—C14—N9	58.7 (6)	O15—N21—C52—C51	−168.6 (6)
C16—N12—C15—N9	59.6 (6)	O14—N21—C52—C51	12.8 (8)
C18—N12—C15—N9	−58.0 (6)	C51—C52—C53—C54	0.1 (9)
C14—N9—C15—N12	58.4 (6)	N21—C52—C53—C54	−178.5 (5)
C13—N9—C15—N12	−59.7 (6)	C52—C53—C54—C49	2.6 (9)
C15—N12—C16—N10	−58.5 (6)	O13—C49—C54—C53	178.4 (5)
C18—N12—C16—N10	58.7 (6)	C50—C49—C54—C53	−3.1 (9)
C13—N10—C16—N12	57.1 (6)	O16—C55—C56—C57	178.0 (5)
C17—N10—C16—N12	−59.0 (6)	C60—C55—C56—C57	−2.6 (9)
C18—N11—C17—N10	−59.4 (6)	C55—C56—C57—C58	1.5 (9)
C14—N11—C17—N10	58.5 (6)	C56—C57—C58—C59	0.7 (9)
C13—N10—C17—N11	−57.1 (6)	C56—C57—C58—N22	−176.8 (5)
C16—N10—C17—N11	59.1 (6)	O17—N22—C58—C57	6.9 (8)
C17—N11—C18—N12	58.4 (7)	O18—N22—C58—C57	−171.7 (5)
C14—N11—C18—N12	−60.0 (7)	O17—N22—C58—C59	−170.8 (6)
C16—N12—C18—N11	−57.7 (7)	O18—N22—C58—C59	10.7 (8)
C15—N12—C18—N11	60.0 (7)	C57—C58—C59—C60	−1.9 (9)
C20—N13—C19—N14	−58.3 (7)	N22—C58—C59—C60	175.7 (5)
C21—N13—C19—N14	60.0 (6)	C58—C59—C60—C55	0.7 (9)
C22—N14—C19—N13	57.6 (6)	O16—C55—C60—C59	−179.1 (5)
C23—N14—C19—N13	−58.5 (6)	C56—C55—C60—C59	1.5 (9)
C19—N13—C20—N15	58.0 (7)	O19—C61—C62—C63	177.9 (5)
C21—N13—C20—N15	−59.7 (6)	C66—C61—C62—C63	−3.7 (8)
C22—N15—C20—N13	−58.1 (6)	C61—C62—C63—C64	3.2 (9)
C24—N15—C20—N13	59.2 (6)	C62—C63—C64—C65	−0.8 (9)
C20—N13—C21—N16	59.1 (6)	C62—C63—C64—N23	−177.9 (5)
C19—N13—C21—N16	−59.5 (6)	O20—N23—C64—C63	6.7 (9)
C23—N16—C21—N13	58.6 (6)	O21—N23—C64—C63	−171.7 (5)
C24—N16—C21—N13	−58.1 (6)	O20—N23—C64—C65	−170.5 (6)
C20—N15—C22—N14	58.8 (6)	O21—N23—C64—C65	11.1 (8)
C24—N15—C22—N14	−58.2 (6)	C63—C64—C65—C66	−1.2 (9)
C19—N14—C22—N15	−58.3 (6)	N23—C64—C65—C66	175.9 (5)
C23—N14—C22—N15	58.0 (6)	C64—C65—C66—C61	0.8 (9)
C24—N16—C23—N14	59.5 (6)	O19—C61—C66—C65	−179.9 (5)
C21—N16—C23—N14	−57.5 (6)	C62—C61—C66—C65	1.6 (8)
C22—N14—C23—N16	−58.9 (6)	O22—C67—C68—C69	−179.2 (5)
C19—N14—C23—N16	57.1 (6)	C72—C67—C68—C69	0.5 (9)
C23—N16—C24—N15	−59.0 (6)	C67—C68—C69—C70	1.4 (9)
C21—N16—C24—N15	58.6 (6)	C68—C69—C70—C71	−1.3 (9)
C22—N15—C24—N16	58.6 (6)	C68—C69—C70—N24	175.3 (5)
C20—N15—C24—N16	−58.8 (6)	O24—N24—C70—C71	8.9 (9)
O1—C25—C26—C27	−178.8 (6)	O23—N24—C70—C71	−169.7 (5)
C30—C25—C26—C27	3.4 (10)	O24—N24—C70—C69	−167.9 (6)
C25—C26—C27—C28	−1.7 (9)	O23—N24—C70—C69	13.5 (8)
C26—C27—C28—C29	0.7 (9)	C69—C70—C71—C72	−0.7 (9)
C26—C27—C28—N17	177.9 (5)	N24—C70—C71—C72	−177.3 (5)
O3—N17—C28—C27	179.2 (6)	C70—C71—C72—C67	2.6 (9)
O2—N17—C28—C27	−0.8 (8)	O22—C67—C72—C71	177.1 (6)
O3—N17—C28—C29	−3.5 (9)	C68—C67—C72—C71	−2.6 (9)

### Hydrogen-bond geometry (Å, °)

**Table:**

*D*—H···*A*	*D*—H	H···*A*	*D*···*A*	*D*—H···*A*
O1—H1O···O1W	0.84	1.75	2.574 (7)	169
O4—H4O···O2W	0.84	1.76	2.585 (7)	168
O7—H7O···O3W	0.84	1.76	2.584 (7)	168
O10—H10O···O4W	0.84	1.76	2.584 (7)	168
O13—H13O···N2	0.84	1.81	2.649 (6)	173
O16—H16O···N6	0.84	1.81	2.641 (6)	170
O19—H19O···N10	0.84	1.82	2.648 (7)	170
O22—H22O···N14	0.84	1.81	2.645 (7)	171
O1W—H1W1···N1	0.84	2.01	2.814 (8)	161
O1W—H1W2···N4^i^	0.84	2.02	2.851 (7)	169
O2W—H2W1···N5	0.84	2.02	2.837 (7)	166
O2W—H2W2···N7^ii^	0.84	1.98	2.811 (7)	172
O3W—H3W1···N9	0.84	2.04	2.830 (8)	158
O3W—H3W2···N11^ii^	0.84	2.01	2.845 (7)	170
O4W—H4W1···N13	0.84	2.03	2.832 (7)	161
O4W—H4W2···N16^i^	0.84	1.99	2.819 (7)	171

Symmetry codes: (i) *x*+1, *y*, *z*; (ii) *x*−1, *y*, *z*.
